# Effects of different doses of remimazolam on the quality of sedation and cardiac function in elderly patients: a double-blind randomised controlled study

**DOI:** 10.3389/fcvm.2024.1453608

**Published:** 2025-01-06

**Authors:** Liu Minghong, Qizhu Feng, Huichun Chen, Ju Li, Jun Shi

**Affiliations:** ^1^ Department of Anesthesiology, The First Affiliated Hospital of Anhui University of Science and Technology, Huainan, Anhui, China; ^2^ Department of General Surgery, The First Affiliated Hospital of Anhui University of Science and Technology, Huainan, Anhui, China; ^3^ Department of Laboratory Medicine, The First Affiliated Hospital of Anhui University of Science and Technology, Huainan, Anhui, China

**Keywords:** remimazolam, sedation, cardiac function, cardiac output, peripheral vascular resistance

## Abstract

**Background:**

We intended to observe the effects of different doses of remimazolam besylate via intravenous induction on the quality of sedation and cardiac function in elderly patients.

**Methods:**

A total of 135 ASA I–III patients undergoing elective laparoscopic cholecystectomy were selected. They were divided into three groups and they were randomized. Low dose group (Group L): Remimazolam besylate 0.2 mg/kg; middle dose group (Group M): Remimazolam besylate 0.3 mg/kg; High dose group (Group H): Remimazolam besylate 0.4 mg/kg. There were 45 patients in each group. The blood pressure, heart rate, BIS values, cardiac function before induction (T_0_), after induction (T_1_) and after intubation (T_2_), as well as the length of loss of consciousness, duration of sedation, and extubation and adverse events were recorded.

**Results:**

At T_1_ and T_2_, systolic and diastolic blood pressure in Group M were lower than those in Group L, but higher than those in Group H, with statistically significant differences (*P* < 0.05). At T_1_ and T_2_, the BIS value in Group M was lower than that in Group L, with a statistically significant difference (*P* < 0.05). However, there was no statistically significant difference (*P* *>* 0*.*05) in BIS values between Group M and Group H; At T_1_ and T_2_, the cardiac output and stroke volume in Group M were higher than those in Group H, while the systemic vascular resistance in Group M was lower than that in Group H, with statistically significant differences (*P* < 0.05); The incidences of bucking when moving and hiccup in Group L were higher than those in Group M and Group H, with statistically significant differences (*P* < 0.05). The number of vasoactive drugs used in Group H was higher than that in Group L and Group M, with statistically significant differences (*P* < 0.05).

**Conclusions:**

General anesthesia induction with remimazolam besylate at 0.3 mg/kg in elderly patients undergoing laparoscopic cholecystectomy showed good quality of sedation, could achieve rapid intubation, with minimal effect on cardiac function and generally favorable safety profile.

## 1 Introduction

Remimazolam besylate, a new benzodiazepine, is an ultra-short-acting gamma-aminobutyric acid A receptor agonist with combined advantages of propofol and midazolam (1). The drug shows great promise as an induction agent due to its favorable phramacokinetic (2). remimazolam is a rapidly hydrolyzable ester-based side chain added to the structure of midazolam. Therefore, it exhibits similar pharmacokinetic characteristics to remifentanil (rapid onset of action and rapid metabolism).

The usual mode of administration in clinical practice is continuous intravenous infusion. Compared with slow continuous infusion, a single rapid intravenous injection of remimazolam simplifies the anesthesia induction process, reduces the waiting time for patients during the administration of anesthetic drugs, precisely controls the dose of each administration, and is more suitable for a rapid induction process in the operating room. This method of administration has been used in painless gastroenteroscopy, general anesthesia for patients with hepatic and renal insufficiency, and even the induction of anesthesia in renal transplant patients (3–5).

Although it has been shown that remimazolam is characterized by rapid onset of action and recovery, further studies are needed to investigate the effects of its use in elderly patients, especially in terms of cardiac function effects. The purpose of this study was to investigate the effects of different doses of remimazolam besylate via induction on the quality of sedation and cardiac function in elderly patients by a prospective randomized controlled study.

## 2 Methods

### 2.1 Included subjects

This study has been approved by the Medical Ethics Committee of the hospital (2021-Lun Shen-030). All patients agreed to participate in the experiment and signed informed consent. Our study subjects were 135 patients hospitalized in the First Affiliated Hospital of Anhui University of Science and Technology, China from March 2021 to March 2023.

### 2.2 Inclusion criteria

(1) ASA physical status I–III (2) Inpatients undergoing elective laparoscopic cholecystectomy (3) Operation time ≤120 min (4) Voluntary participation and signed informed consent. (5) Age ≥65 years. (6) BMI ≤30 kg/m^2^. (7) Blood pressure ≤160/100 mmHg. Exclusion criteria: (1) Patients with mental illness; (2) Severe communication and expression disorders; (3) BMI > 30 kg/m^2^; (4) Use of sedatives or analgesics 24 h before surgery; (5) Participated in other drug trials within 3 months. (6) Age <65 years. (7) Patients with systolic blood pressure ≥180 mmHg and/or diastolic blood pressure ≥110 mmHg. (The blood pressure standard is based on the definition of severe hypertension in the 2024 revised version of the Chinese Hypertension Prevention and Treatment Guidelines.) Such exclusion criteria are intended to ensure the safety of the research subjects, as extreme hypertension may increase the risk of cardiovascular and cerebrovascular complications during surgery. Exclusion criteria: (1) Patients withdraw their informed consent (2) Patients who do not meet the inclusion criteria or meet the exclusion criteria (3) Lost to follow-up. No statistical analysis of efficacy will be performed for excluded cases.

### 2.3 Sample size calculation

The sample size was calculated based on a preliminary experiment of 40 people in our research center. In the preliminary experiment, the incidence of hypotension in the L group was 5.1%, and that in the H group was 24.5%, and the difference was statistically significant (*P* < 0.05). Considering that there is no randomized controlled study comparing the two concentrations, the required sample size for each group was at least 41 people, according to the results of the previous study of our research center, based on *α* = 0.05 (bilateral), 1-*β* = 0.8, and a ratio of 1:1, calculated by PASS 15.0 (NCSS, USA) software. Considering the 10% loss to follow-up rate, the sample size of each group was increased to 45 people. A total of 150 patients were evaluated for eligibility, of whom 12 were excluded for not meeting the inclusion criteria, 1 refused to participate, and 2 were lost to follow-up. The final sample size was determined to be 45 cases per group.

### 2.4 Grouping

Patients were divided into three groups by the random number method: Low dose group (Group L): Remimazolam besylate 0.2 mg/kg; middle dose group (Group M): Remimazolam besylate 0.3 mg/kg; High dose group (Group H): Remimazolam besylate 0.4 mg/kg. There was no statistically significant difference (*P* > 0.05) in general data such as age, body mass, blood loss, operation duration and anesthesia duration among the three groups, with comparability (see Table 1).

**Table 1:** Comparison of general data $\left(\bar{x}\pm s\right)$.

Group	Number of cases	Age (years)	ASA condition (II/III)	Body mass (kg/m^2^)	Blood loss (ml)	Operation duration (min)	Anesthesia duration (min)	Sufentanil induction medication (ug)
Group L	45	68.5 ± 5.2	30/15	23.9 ± 5.1	4.6 ± 2.0	59.2 ± 12.7	65.8 ± 8.6	31.0 ± 3.4
Group M	45	68.7 ± 5.1	32/13	24.9 ± 5.9	4.7 ± 1.9	56.3 ± 13.4	67.6 ± 8.3	30.2 ± 3.9
Group H	45	68.8 ± 5.0	29/16	25.1 ± 4.4	4.5 ± 2.1	57.8 ± 13.1	64.1 ± 9.0	29.8 ± 4.1

## 3 Methods

Patients fasted from solid food for 8 h and 4 h from clear fluids before anaesthesia. After admission to the operating room, the peripheral venous access of the upper limb was established and Lactated Ringer's Solution was infused at a rate of 5 ml/kg/h. Noninvasive blood pressure (NIBP), 6-lead electrocardiogram (ECG), oxygen saturation (SpO_2_) and depth of anesthesia (BIS) were monitored. Clean the skin of the patient's neck and chest, symmetrically stick disposable ECG electrodes on the patient's bilateral sternocleidomastoid muscles and mid-axillary line (xiphoid level), and connect the device to a non-invasive hemodynamic detector (model: BioZ-2011-101, Shenzhen Madean Medical Equipment Co.). Monitor cardiac function-related parameters (cardiac output, stroke volume, systemic vascular resistance, and myocardial acceleration index) during anesthesia induction. The rate of oxygen inhalation through the nasal cannula was 3 L/min, and anesthesia began after pre-oxygen inhalation for 3 min. The intravenous anesthesia was adopted: intravenous bolus of remimazolam besylate (1 mg/ml): Low dose group: 0.2 mg/kg; middle dose group: 0.3 mg/kg; High dose group: 0.4 mg/kg. When the patient gradually loses consciousness, the BIS value gradually decreases. When the BIS value stops decreasing and rebounds, the previous value is the lowest BIS value, and this BIS value is recorded. Sufentanil (0.5 µg/kg) and rocuronium (0.7 mg/kg) were added after the patient lost consciousness and the BIS value decreased to the lowest level. Tracheal intubation was performed after the BIS value decreased to 60 after the onset of muscle relaxation. If the blood pressure dropped to 90/60 mmHg, 6 mg of ephedrine was given once; If the heart rate dropped to 50 beats/min, 0.3 mg of atropine was given once. During the operation, propofol and remifentanil were continuously infused via intravenous pump to maintain the BIS value within the range of 40–60, and additional drugs were added according to the metabolic time of rocuronium bromide. After the operation, when the patient's breathing was recovered and regular, oxygen inhalation has been stopped for 5 min and oxygen saturation continued to exceed 95%, extubation was performed, and the patient was connected with an intravenous analgesia pump and returned to the PACU for observation for 30 min.

### 3.1 Evaluation indicators

1. Evaluation of sedation quality: before induction (T0), i.e., after the patient enters the operating room, is in a quiet state, establishes intravenous access, and is ready to start induction; before intubation (T1), i.e., exposing the glottis and preparing for intubation; after intubation (T2), i.e., after intubation, the catheter is fixed, and machine-controlled breathing is 3 min later. At these time points, the patient's blood pressure, heart rate, and BIS value (COVIDIEN bispectral index meter) were measured;
2. Cardiac function monitoring: The BioZ-2011-101 non-invasive hemodynamic monitor was used to monitor cardiac output, stroke volume, peripheral vascular resistance (SVR) and myocardial acceleration index (ACI); data were observed and recorded at three time points: before induction (T0), before intubation (T1) and after tracheal intubation (T2). The monitoring results were compared with the patient's baseline data to evaluate the effects of anesthetic drugs on cardiac function. Non-invasive hemodynamic monitors provide a non-invasive way to monitor hemodynamic parameters, which is more comfortable for patients and reduces the risks of invasive procedures. Non-invasive hemodynamic monitors can provide real-time hemodynamic data, which is essential for quickly assessing the patient's blood flow status. Although non-invasive monitoring devices may differ from traditional invasive monitoring devices in accuracy, non-invasive hemodynamic monitors have been shown in multiple clinical studies to provide reliable hemodynamic monitoring data. For example, in the study by Ying-Kuang Lin et al. (6), non-invasive hemodynamic monitoring was used to assess the hemodynamic status of hemodialysis patients, and the results showed that the device could provide data comparable to invasive monitoring. The meta-analysis by Peyton PJ and Chong SW (7) also confirmed the accuracy and precision of non-invasive hemodynamic monitoring in surgery and intensive care;
3. Loss of consciousness time: from the completion of intravenous injection of remimazolam besylate to the patient falling asleep, losing eyelash reflex and verbal response in the state of loss of consciousness, record the duration and the lowest BIS value during the period (8);
4. Intubation time: from the end of the injection of the induction drug (the last rocuronium bromide) to the time when the BIS value drops to 60, the patient's lower jaw relaxes, the electronic laryngoscope is inserted, the glottis is clearly exposed, and the endotracheal tube is ready to be inserted;
5. Extubation time: refers to the time from the cessation of intravenous infusion of remifentanil and propofol to suctioning and removal of the endotracheal tube;
6. Adverse events: List all adverse events observed during the study, including: coughing due to body movement, use of vasoactive drugs, respiratory depression, nausea and vomiting, postoperative delirium, hiccups, etc.

These evaluation indicators are used to measure the effects of different doses of remimazolam besylate on the quality of sedation and cardiac function of patients. Through these indicators, the effect of anesthetic drugs and their impact on the patient's physiological state can be evaluated.

### 3.2 Statistical analysis

SPSS 26.0 statistical software was used for data analysis, measurement data were expressed as $\left(\bar{x}\pm s\right)$, the paired *t*-test was used for comparison between groups. Enumeration data were expressed as *n* (%), and the *χ*^2^ test was used for comparison between groups; Differences were considered statistically significant if *P* < 0.05.

## 4 Results

### 4.1 General information

All patients were aged between 65 and 85 years, with an average age of 74.4 ± 9.6 years. The body mass index (BMI) of the patients ranged from 18.5 to 30 kg/m², with an average of 24.6 ± 5.2 kg/m². Gender distribution: 67 males and 68 females. The ASA physical condition of all patients was II–III. All patients underwent laparoscopic cholecystectomy. The average operation time was 57.7 ± 13.1 min. The average blood loss was 4.6 ± 2.0 ml. The average dose of sufentanil during induction: 30.2 ± 3.9 ug. The three groups of patients were compared in terms of demographic data such as age, body weight, blood loss, operation time, and anesthesia time. There was no significant difference (*P* > 0.05), and the three groups were comparable (see Table 1).

### 4.2 Comparison of quality of sedation

**Figure 1:**
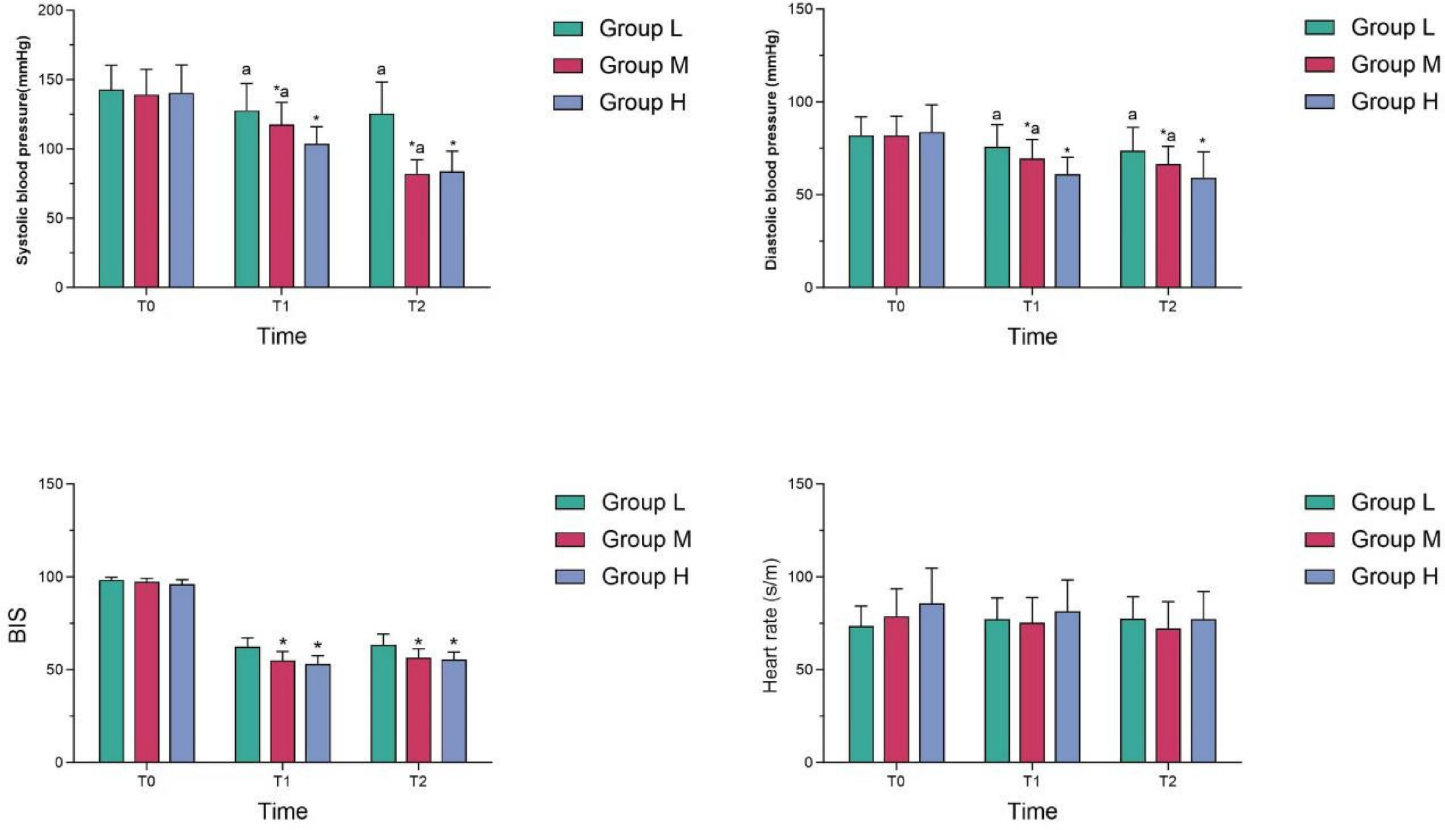
Comparison of quality of sedation among the three groups at different time points.

At T_1_ and T_2_, systolic and diastolic blood pressure in Group M were lower than those in Group L, but higher than those in Group H, and the differences were statistically significant (*P* < 0.05); At T_1_ and T_2_, the BIS value in Group M was lower than that of Group L, and the difference was statistically significant (*P* < 0.05); However, there was no statistically significant difference in BIS between Group M and Group H (*P* > 0.05). There was no statistically significant difference in heart rate among the three groups at different time points (see Table 2 and Figure 1).

**Table 2:** Comparison of quality of sedation among the three groups at different time points $\left(\bar{x}\pm s\right)$.

Group	Number of cases	Systolic blood pressure (mmHg)			Diastolic blood pressure (mmHg)			BIS			Heart rate (s/m)		
		T0	T1	T2	T0	T1	T2	T0	T1	T2	T0	T1	T2
Group L	45	142.8 ± 17.7	127.7 ± 19.5**	125.65 ± 22.64**	82.1 ± 9.8	75.9 ± 11.8**	73.8 ± 12.5**	98.4 ± 1.5	62.4 ± 4.8	63.4 ± 5.8	73.4 ± 10.9	77.3 ± 11.4	77.6 ± 11.7
Group M	45	139.1 ± 18.3	117.6 ± 15.9*^,^ **	111.75 ± 13.73*^,^ **	81.9 ± 10.5	69.5 ± 10.3*^,^ **	66.5 ± 9.6*^,^ **	97.5 ± 1.8	54.9 ± 4.8*	56.6 ± 4.6*	78.7 ± 14.9	75.4 ± 13.6	72.1 ± 14.5
Group H	45	140.4 ± 20.3	103.8 ± 12.32*	101.11 ± 16.0*	83.7 ± 14.7	61.0 ± 9.2*	59.2 ± 13.9*	96.1 ± 2.4	53.1 ± 4.5*	55.6 ± 3.9*	85.7 ± 19.0	81.4 ± 16.8	77.3 ± 14.8

* *P* *<* *0.05* compared with Group L

** *P* *<* *0.05* compared with Group H.

### 4.3 Changes in cardiac function

**Figure 2:**
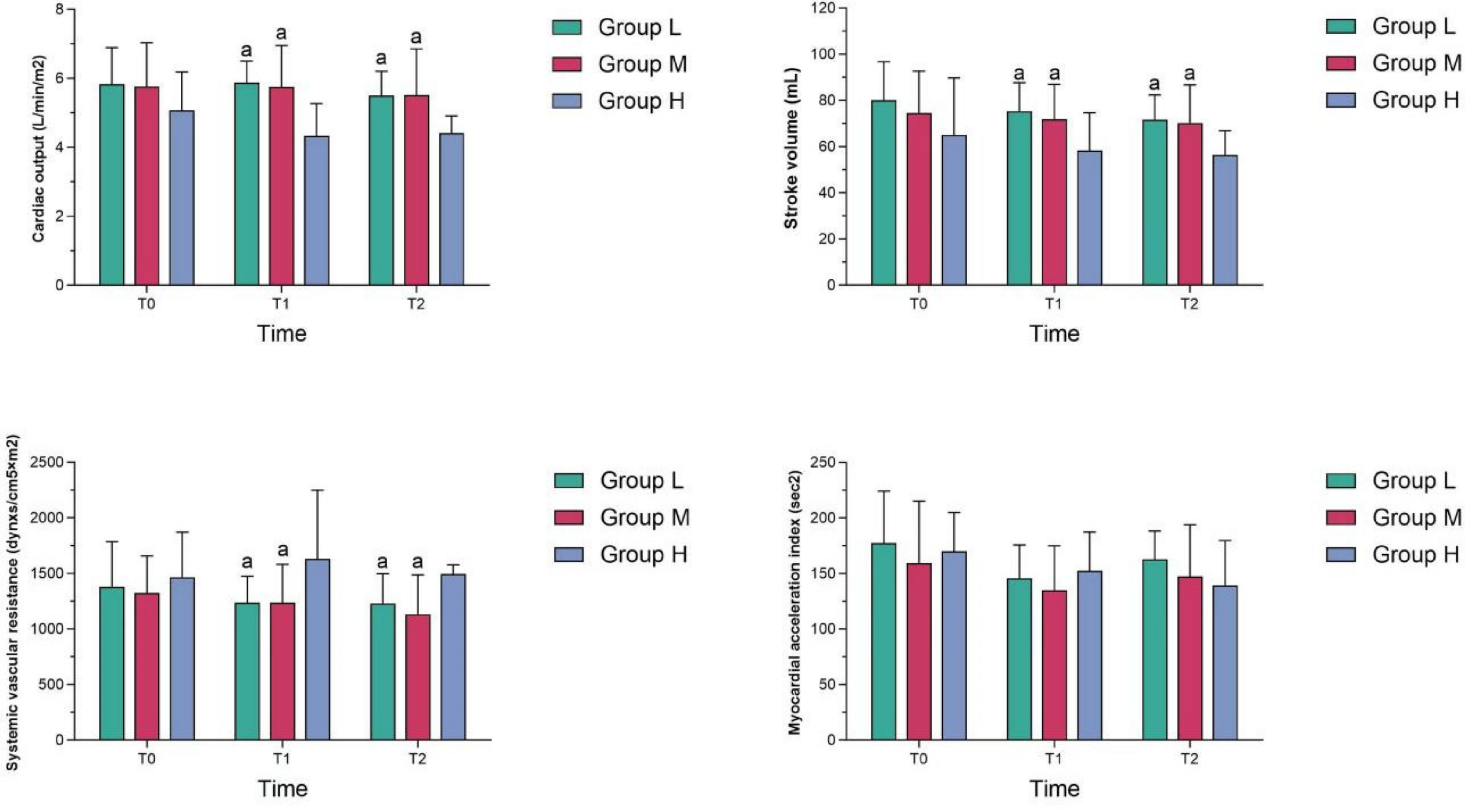
Comparison of cardiac function among the three groups.

At T_1_ and T_2_, the cardiac output and stroke volume in Group M were higher than those in Group H, while the systemic vascular resistance in Group M was lower than that in Group H, and the differences were statistically significant (*P* < 0.05); At T_1_ and T_2_, the cardiac output, stroke volume and systemic vascular resistance in Group M were not statistically different from those in Group L (*P* > 0.05); At T_1_ and T_2_, the myocardial acceleration index in group M was not statistically different from those in the other two groups (*P* > 0.05) (see Table 3 and Figure 2).

**Table 3:** Comparison of cardiac function among the three groups $\left(\bar{x}\pm s\right)$.

Group	Number of cases	Cardiac output (L/min/m^2^)		Stroke volume (ml)				Systemic vascular resistance (dynxs/cm^5^ × m^2^)			Myocardial acceleration index (s^2^)		
		T0	T1	T2	T0	T1	T2	T0	T1	T2	T0	T1	T2
Group L	45	5.8 ± 1.1	5.9 ± 0.6**	5.5 ± 0.7**	80.0 ± 16.8	75.2 ± 12.5**	71.5 ± 10.8**	1,379.9 ± 407.4	1,236.9 ± 237.8**	1,230.6 ± 267.1**	177.2 ± 46.8	145.8 ± 29.8	162.8 ± 25.4
Group M	45	5.7 ± 1.3	5.8 ± 1.2**	5.5 ± 1.3**	74.6 ± 18.1	71.9 ± 15.1**	70.2 ± 16.5**	1,322.4 ± 334.4	1,238.7 ± 343.9**	1,131.9 ± 354.2**	159.4 ± 55.7	134.9 ± 39.7	147.3 ± 46.4
Group H	45	5.0 ± 1.1	4.3 ± 0.9	4.4 ± 0.5	64.9 ± 24.8	58.3 ± 16.3	56.4 ± 10.5	1,465.0 ± 407.4	1,632.5 ± 616.1	1,494.6 ± 384.5	169.8 ± 35.1	152.5 ± 34.9	139.3 ± 40.3

** *P* *<* *0.05* compared with Group H.

### 4.4 Comparison of durations

**Figure 3:**
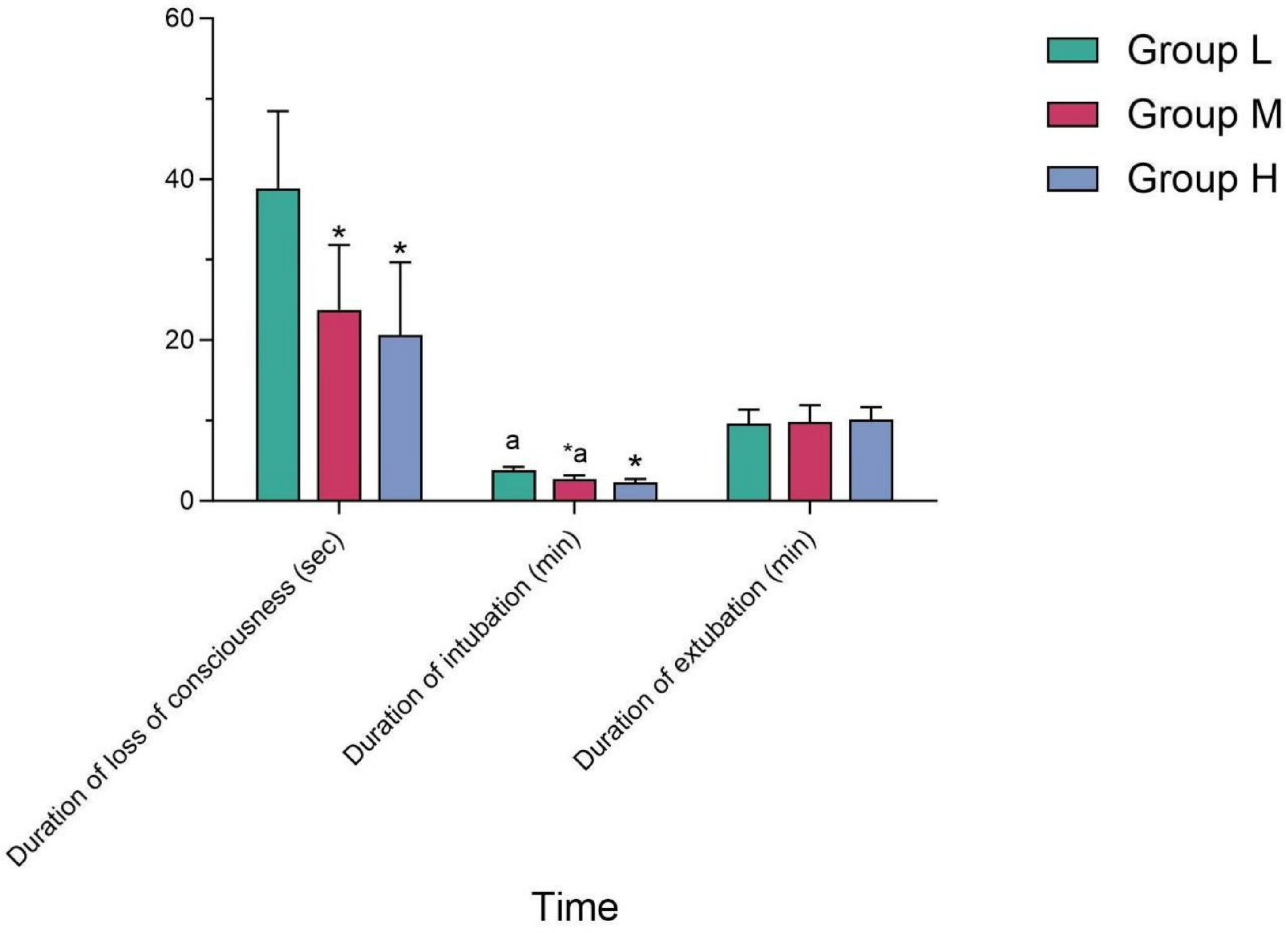
Comparison of durations among the three groups.

The duration of intubation in Group M was longer than that in Group H, and the difference was statistically significant (*P* < 0.05); while compared with Group L, the difference was not statistically significant (*P* > 0.05). There was no statistically significant difference in the duration of sedation and extubation among the three groups (*P* > 0.05) (see Table 4 and Figure 3).

**Table 4:** Comparison of durations among the three groups $\left(\bar{x}\pm s\right)$.

Group	Number of cases	Duration of loss of consciousness (sec)	Duration of intubation (min)	Duration of extubation (min)
Group L	45	38.8 ± 9.6	3.7 ± 0.4**	9.6 ± 1.8
Group M	45	23.7 ± 8.1*	2.7 ± 0.5*^,^ **	9.8 ± 2.1
Group H	45	20.6 ± 9.0*	2.3 ± 0.5*	10.1 ± 1.6

* *P* *<* *0.05* compared with Group.

** *P* *<* *0.05* compared with Group H.

### 4.5 Occurrence of adverse reactions

**Figure 4:**
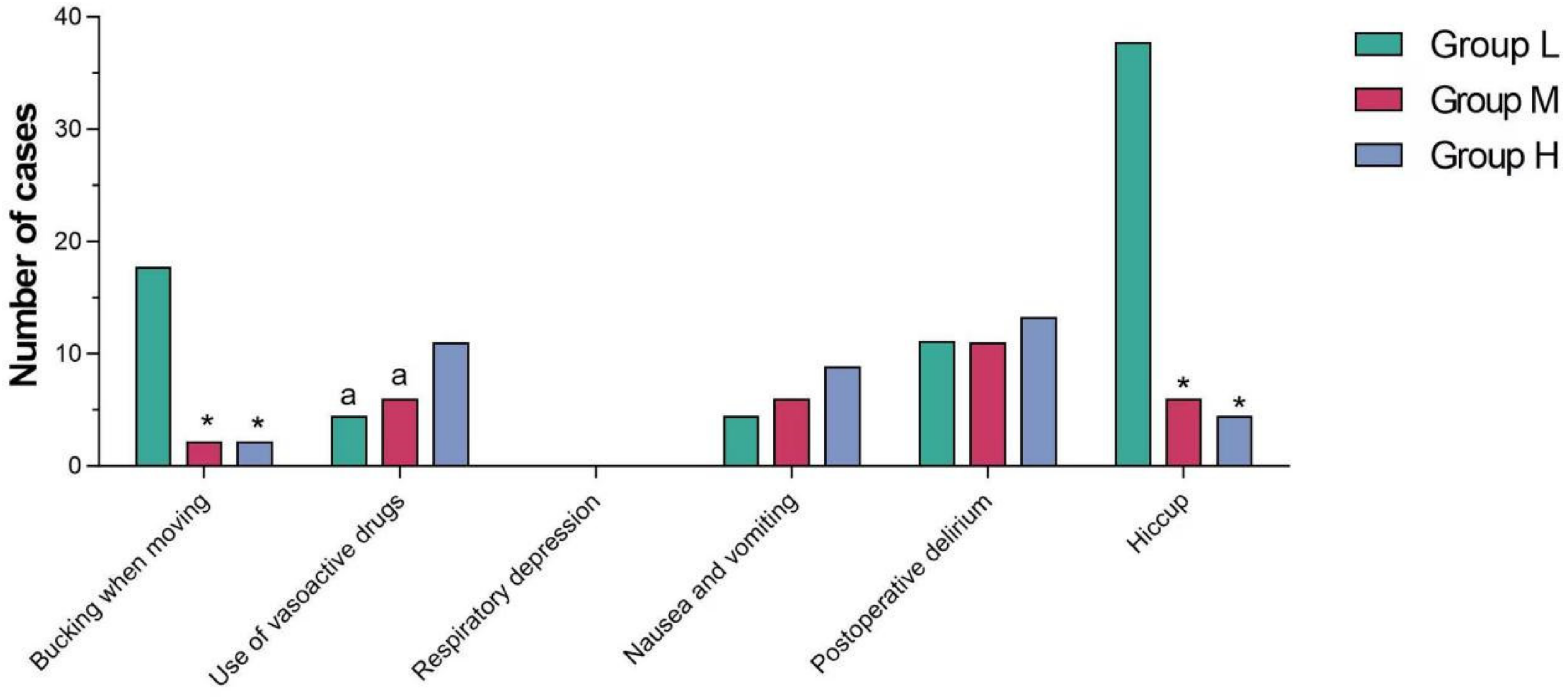
Comparison of adverse reactions among the three groups.

The incidence of bucking when moving and hiccup in Group L was higher than that in Group M and Group H, and the differences were statistically significant (*P* < 0.05). The number of vasoactive drugs used in Group H was higher than that in Group L and Group M, and the differences were statistically significant (*P* < 0.05). There was no significant difference in respiratory depression, nausea and vomiting and postoperative delirium among the three groups (*P* *>* 0.05) (see Table 5 and Figure 4).

**Table 5:** Comparison of adverse reactions among the three groups [*n* (%)].

Group	Number of cases	Bucking when moving	Use of vasoactive drugs	Respiratory depression	Nausea and vomiting	Postoperative delirium	Hiccup
Group L	45	8 (17.77%)	2 (4.44%)**	0 (0.00%)	2 (4.44%)	5 (11.11%)	17 (37.77%)
Group M	45	1 (2.22%)*	3 (6.00%)**	0 (0.00%)	3 (6.00%)	5 (11.11%)	3 (6.00%)*
Group H	45	1 (2.22%)*	11 (24.44%)	0 (0.00%)	4 (8.88%)	6 (13.33%)	2(4.44%)*

* *P* *<* *0.05* compared with Group L.

** *P* *<* *0.05* compared with Group H.

## 5 Discussion

Remimazolam besylate is a new ultra-short-acting benzodiazepine sedative. It stimulates the gamma-aminobutyric acid *α* receptor, opens the chloride channel and promotes chloride influx, which leads to the hyperpolarization of the nerve cell membrane and inhibition of neurons, thus resulting in sedation and amnesia. With the combined advantages of midazolam and remifentanil, it has a rapid onset of action, rapid recovery, with minimal impact on liver and kidney function (9), and is one of the ideal drugs for intravenous general anesthesia.

Remimazolam has fewer effects on heart rate variability than propofol during induction of general anesthesia and maintains a balance between sympathetic and parasympathetic activities (10). We observed during the experiment that there was no significant difference in heart rate changes among the three groups after induction with different doses of remimazolam besylate, which was consistent with the results reported in the previous literature; Compared with propofol, remimazolam demonstrates favorable sedative effects and is more hemodynamically stable (11, 12). Continuous intravenous infusion with a micro-pump is often used clinically for induction of general anesthesia (13, 14). However, the onset time of sedation is longer. Intravenous bolus was used in this experiment, which resulted in a significantly shorter administration time compared with intravenous infusion with a pump. The greater the dose of remimazolam besylate used, the shorter the time to loss of consciousness, the lower the BIS value, the greater the depth of sedation, and the shorter the duration of tracheal intubation. However, when the induction dose of remimazolam besylate was increased to 0.4 mg/kg, the onset time and depth of sedation did not change significantly, while the systolic and diastolic blood pressure decreased significantly, with significant fluctuations in hemodynamics. Monitoring of cardiac function with a non-invasive hemodynamic detector revealed that the systemic vascular resistance of the patient would suddenly increase and the stroke volume would significantly decrease, while the heart rate showed no significant change; the cardiac output would also significantly decrease, but the myocardial acceleration index, which reflects the systolic function of the heart, showed no significant change. Therefore, we speculate that high doses of remimazolam besylate increase cardiac afterload by increasing peripheral circulatory resistance, resulting in decreased stroke volume and decreased cardiac output, with no significant effect on systolic function of the heart. Changes in blood pressure are closely related to cardiac output and systemic vascular resistance (15). Changes in systemic vascular resistance affect changes in cardiac output (7, 16). It has been demonstrated in the literature that stroke volume and cardiac output increase when systemic vascular resistance decreases (17). The sudden increase in systemic vascular resistance may be related to the sudden contraction of peripheral vessels. It has been shown that high doses of remimazolam increase intracellular calcium concentration in a dose-dependent manner (18). Whether this is related to the sudden increase in systemic vascular resistance due to the sudden contraction of peripheral vessels needs to be further verified. Among the adverse reactions, we observed that the BIS value was higher, the depth of sedation was not enough, and that bucking when moving and hiccup often occurred during inductions with low doses of remimazolam besylate; during inductions with high doses of remimazolam besylate, the patient's blood pressure fluctuated drastically, often requiring vasoactive drugs for regulation; whereas moderate doses of remimazolam besylate can achieve a satisfactory depth of sedation without significant hemodynamic changes.

In conclusion, induction with intravenous bolus of remimazolam besylate at 0.3 mg/kg allows rapid achievement of a satisfactory depth of sedation, with minimal effect on cardiac function, stable hemodynamics and few adverse reactions in elderly patients.

However, our study is not without limitations. One notable constraint is that while remimazolam was utilized during the induction phase of anesthesia, propofol was administered during the maintenance phase, with no further use of remimazolam. This approach may have influenced outcomes related to the quality of patient emergence from anesthesia and cardiovascular stability, potentially obscuring any distinct effects attributable to remimazolam in these domains. Furthermore, the limited sample size and the fact that the study was conducted at a single medical institution introduce the risk of selection bias and may reflect specific practice patterns that limit the generalizability of our findings.

## 6 Conclusions

In conclusion, induction with intravenous bolus of remimazolam besylate at 0.3 mg/kg allows rapid achievement of a satisfactory depth of sedation, with minimal effect on cardiac function, stable hemodynamics and few adverse reactions in elderly patients.

## Data Availability

The datasets presented in this study can be found in online repositories. The names of the repository/repositories and accession number(s) can be found in the article/Supplementary Material.
